# Epidemiology of congenital malformations of the external ear in Hunan Province, China, from 2016 to 2020

**DOI:** 10.1097/MD.0000000000037691

**Published:** 2024-04-12

**Authors:** Xu Zhou, Junqun Fang, Xiaoli Wang, Haiyan Kuang, Jian He, Aihua Wang, Xinjun Hua, Xiu Zeng, Shuxian Zeng

**Affiliations:** a Hunan Provincial Maternal and Child Health Care Hospital, Changsha, Hunan Province, China.

**Keywords:** anotia, congenital abnormalities, ear malformations, epidemiology, microtia, prevalence

## Abstract

To describe the epidemiology of congenital malformations of the external ear (CMEE). Data were obtained from the Birth Defects Surveillance System in Hunan Province, China, 2016 to 2020. The prevalence of CMEEs is defined as the number of cases per 1000 fetuses (births and deaths at 28 weeks of gestation and beyond) (unit: ‰). Prevalence and 95% confidence intervals (CI) were calculated by the log-binomial method. Chi-square trend tests (*χ^2^*_*trend*_) were used to determine trends in prevalence by year. *P* < .05 was considered statistically significant. Crude odds ratios (ORs) were calculated to examine the association of sex, residence, and maternal age with CMEEs. Our study included 847,755 fetuses, and 14,459 birth defects were identified, including 1227 CMEEs (accounted for 8.49% of birth defects). The prevalences of birth defects and CMEEs were 17.06‰ (95%CI: 16.78–17.33) and 1.45‰ (95%CI: 1.37–1.53), respectively. A total of 185 microtia-anotias were identified, accounting for 15.08% of CMEEs, with a prevalence of 0.22‰ (95%CI: 0.19–0.25). And 1042 other CMEEs were identified, accounting for 84.92% of CMEEs. From 2016 to 2020, the prevalences of birth defects were 18.20‰, 18.00‰, 16.31‰, 16.03‰, and 16.47‰, respectively, showing a downward trend (χ^2^_trend_ =8.45, *P* < .01); the prevalences of CMEEs were 1.19‰, 1.62‰, 1.80‰, 1.21‰, and 1.35‰, respectively, with no significant trend (χ^2^_trend_ =0.09, *P* = .77). CMEEs were more common in males than females (1.60‰ vs 1.27‰, OR = 1.26, 95%CI: 1.12–1.41), in urban areas than in rural areas (1.77‰ vs 1.23‰, OR = 1.45, 95%CI: 1.29–1.62). The prevalences of CMEEs for maternal age < 20, 20–24, 25–29, 30–34, and ≥ 35 were 1.75‰, 1.27‰, 1.44‰, 1.47‰, and 1.58‰, respectively, with no significant difference (*P* > .05, reference: 25–29). Most CMEEs were diagnosed by clinical examinations (92.34%), and most CMEEs were diagnosed postpartum (within 7 days) (96.25%). In summary, we have presented the epidemiology of CMEEs in Hunan Province, China. CMEEs were more common in males than females, in urban areas than rural areas, whereas there was no significant difference in prevalence of CMEEs by maternal age. We inferred that CMEEs may be mainly related to genetics, and the mechanism needs to be examined in the future.

## 1. Introduction

Birth defects are structural or functional anomalies at or before birth.^[1]^ The prevalence of birth defects is 2% to 3% worldwide^[2]^ and is estimated to be 4% to 6% in China.^[3]^ Congenital malformation of the external ear (CMEE) is one of the most common birth defects, with a reported prevalence of 1:6000 until 1:6830 newborns.^[4]^ The prevalence of CMEEs was 16.92 per 10,000 perinatal infants in Hunan Province, China (2010–2020).^[5]^ Common CMEEs include microtia-anotias, preauricular tags, and prominent ears.^[4,6]^ Although most CMEEs have minimal physiologic impact, even minor malformations can present an aesthetic and psychosocial concern for pediatric patients and their parents, such as psychological distress, anxiety, social avoidance, and behavioral problems.^[7,8]^ In addition, many CMEEs are combined with middle ear malformations or may be found in syndromes.^[9–11]^ Therefore, studies on CMEEs are significant and deserve more attention.

There were some studies on the classification, genetics, natural history, and management of CMEEs. E.g., Chang et al reviewed the types of deformational ear deformities and the most up-to-date literature on ear molding^[12]^; Daniali et al studied the treatment of ear malformations in newborns^[13]^; Lennon et al studied nonsurgical management of congenital auricular anomalies^[14]^; Klockars et al studied embryology and epidemiology of microtia^[15]^; Alasti et al studied genetics of microtia^[16]^; Bartel-Friedrich et al reviewed syndromes of congenital auricular malformations^[17]^; Siegert et al studied the otoplasty and auricular reconstruction.^[18]^ There were some studies on the epidemiology of CMEEs. E.g., Xie et al reported that the prevalences of microtia-anotias and other CMEEs were 2.22 and 17.39 per 10,000 perinatal infants, respectively, in Hunan Province, China (2005–2014)^[19]^; The prevalence of anotia was 0.04 per 1000 births in Europe (2003–2007)^[20]^; The prevalence of congenital aural atresia and cryptotia were 3.9 and 3.2 per 10,000 pregnancies, respectively, in Japan (2011–2014)^[21]^; The prevalences of anotia and microtia were 0.47 and 2.9 per 10,000 live births in Korea (2008–2014)^[22]^; The prevalence of microtia-anotias was 1.6 per 10,000 births in Kampala, Uganda (2015–2017)^[23]^; Bartel-Friedrich et al found microtia is more frequent in males^[17]^; Zhu et al found the prevalence of anotia and microtia in the urban area was significantly higher than that in the rural area^[24,25]^; Forrester et al found anotia and microtia were more common in infants with advanced maternal age.^[26]^ However, there are limitations in these studies. First, although the prevalence of some specific CMEEs (such as anotia and microtia) has been reported in some studies, to our knowledge, there were almost no systematic in-depth epidemiologic studies on CMEEs. Second, more systematic studies on the epidemiology of CMEEs in China are needed. Third, some previous studies could have been more extensive in data, such as relatively few cases included or surveys conducted in unrepresentative districts or a shorter period. Fourth, some studies needed to be updated.

Therefore, we conducted a comprehensive analysis from hospital-based surveillance in Hunan Province, China, 2016 to 2020, to describe the epidemiology of CMEEs, which may make some original contribution to the field.

## 2. Methods

### 2.1. Data sources

**Figure 1.**
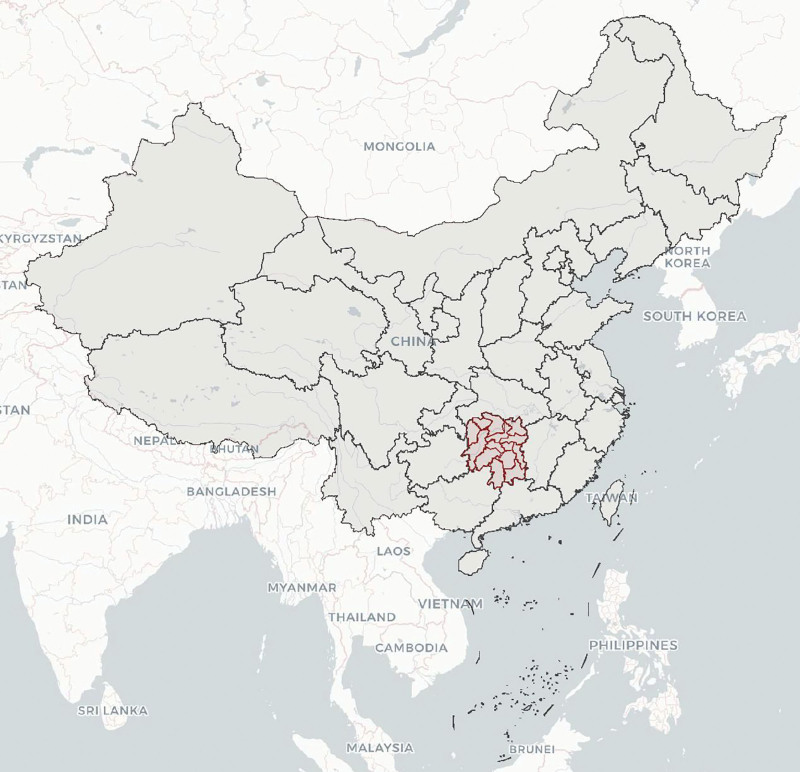
Location of Hunan Province, China.

**Figure 2.**
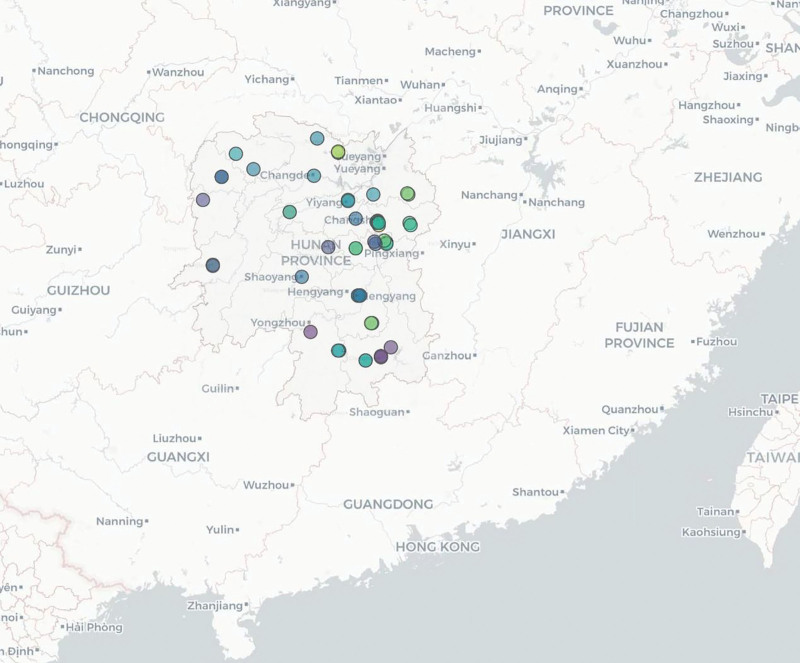
Distribution of birth defects surveillance sites.

The data was derived from the Birth Defects Surveillance System in Hunan Province, China, 2016 to 2020, which is run by the Hunan Provincial Health Commission and involves 52 representative registered hospitals in Hunan Province. Details are presented in Figures 1 and 2. Detailed information on birth defects surveillance has been reported elsewhere.^[3]^ Surveillance data of fetuses (births and deaths at 28 weeks of gestation and beyond) and all birth defects (between 28 weeks of gestation and 7 days after delivery) included demographic characteristics such as sex, residence, maternal age, and other key information.

In the Birth Defects Surveillance System in Hunan Province, CMEEs are classified as anotia, microtia, and other CMEEs. Since severe microtia usually contain anotia,^[27]^ they are classified into the same group (microtia-anotia).

### 2.2. Definitions

The prevalence of CMEEs is defined as the number of cases per 1000 fetuses (births and deaths at 28 weeks of gestation and beyond) (unit: ‰).

### 2.3. Ethics approval and consent to participate

The study was approved by the Hunan Provincial Maternal and Child Health Care Hospital (NO: 2023-S019). It is a retrospective study of medical records; all data were fully anonymized before we accessed them. Moreover, we deidentified the patient records before analysis. We confirmed that all experiments were performed following relevant guidelines and regulations. We confirmed that informed consent was obtained from all subjects and/or their legal guardian(s). Doctors obtain consent from pregnant women before collecting surveillance data, witnessed by their families and the heads of the obstetrics or neonatal departments. Doctors obtain consent from their parents or guardians for live births witnessed by their families and the heads of the obstetrics or neonatal departments. Since the Health Commission of Hunan Province collects those data, and the government has emphasized the privacy policy in the “Maternal and Child Health Monitoring Manual in Hunan Province,” there is no additional written informed consent.

### 2.4. Data quality control

To carry out surveillance, the Hunan Provincial Health Commission formulated the “Maternal and Child Health Monitoring Manual in Hunan Province.” Data were collected and reported by experienced doctors. To reduce the integrity and information error rates of surveillance data, the Hunan Provincial Health Commission asked the technical guidance departments to conduct comprehensive quality control each year.

### 2.5. Statistical analysis

Prevalence and 95% confidence intervals (CI) were calculated by the log-binomial method.^[28]^ Chi-square trend tests (*χ^2^*_*trend*_) were used to determine trends in prevalence by year. *P* < .05 was considered statistically significant. Crude odds ratios (ORs) were calculated to examine the association of sex, residence, and maternal age with CMEEs.

Statistical analyses were performed using SPSS 18.0 (IBM Corp., NY).

## 3. Results

### 3.1. Prevalence of CMEEs in Hunan Province, China, 2016 to 2020

Our study included 847,755 fetuses, and 14,459 birth defects were identified, including 1227 CMEEs (accounted for 8.49% of birth defects). The prevalences of birth defects and CMEEs were 17.06‰ (95%CI: 16.78–17.33) and 1.45‰ (95%CI: 1.37–1.53), respectively. A total of 185 microtia-anotias were identified, accounting for 15.08% of CMEEs, with a prevalence of 0.22‰ (95%CI: 0.19–0.25). And 1042 other CMEEs were identified, accounting for 84.92% of CMEEs.

From 2016 to 2020, the prevalences of birth defects were 18.20‰, 18.00‰, 16.31‰, 16.03‰, and 16.47‰, respectively, showing a downward trend (χ^2^_trend_ =8.45, *P* < .01); the prevalences of CMEEs were 1.19‰, 1.62‰, 1.80‰, 1.21‰, and 1.35‰, respectively, with no significant trend (χ^2^_trend_ =0.09, *P* = .77) (Table 1).

**Table 1:** Prevalence of congenital malformations of the external ear in Hunan Province, China, 2016 to 2020.

Yr	Fetuses (n)	Birth defects (n)		CMEE (n)			Microtia-anotia			Other CMEE		
		n	Prevalence (‰,95%CI)	n	Prevalence (‰,95%CI)	Proportion in birth defects (%)	n	Prevalence (‰,95%CI)	Proportion in CMEE (%)	n	Prevalence (‰,95%CI)	Proportion in CMEE (%)
2016	170,688	3107	18.20 (17.56–18.84)	203	1.19 (1.03–1.35)	6.53	28	0.16 (0.10–0.22)	13.79	175	1.03 (0.87–1.18)	86.21
2017	196,316	3533	18.00 (17.40–18.59)	319	1.62 (1.45–1.80)	9.03	40	0.20 (0.14–0.27)	12.54	279	1.42 (1.25–1.59)	87.46
2018	177,762	2900	16.31 (15.72–16.91)	320	1.80 (1.60–2.00)	11.03	33	0.19 (0.12–0.25)	10.31	287	1.61 (1.43–1.80)	89.69
2019	164,840	2643	16.03 (15.42–16.65)	199	1.21 (1.04–1.37)	7.53	39	0.24 (0.16–0.31)	19.60	160	0.97 (0.82–1.12)	80.40
2020	138,149	2276	16.47 (15.80–17.15)	186	1.35 (1.15–1.54)	8.17	45	0.33 (0.23–0.42)	24.19	141	1.02 (0.85–1.19)	75.81
Total	847,755	14,459	17.06 (16.78–17.33)	1227	1.45 (1.37–1.53)	8.49	185	0.22 (0.19–0.25)	15.08	1042	1.23 (1.15–1.30)	84.92

CMEE = congenital malformations of the external ear.

### 3.2. Prevalence of CMEEs by sex, residence, and maternal age

CMEEs were more common in males than females (1.60‰ vs 1.27‰, OR = 1.26, 95%CI: 1.12–1.41), in urban areas than in rural areas (1.77‰ vs 1.23‰, OR = 1.45, 95%CI: 1.29–1.62). The prevalences of CMEEs for maternal age < 20, 20–24, 25–29, 30–34, and ≥ 35 were 1.75‰, 1.27‰, 1.44‰, 1.47‰, and 1.58‰, respectively, with no significant difference (*P* > .05, reference: 25–29).

Microtia-anotias were more common in males than females (0.26‰ vs 0.17‰, OR = 1.57, 95%CI: 1.16–2.12). The prevalences of microtia-anotias for urban and rural areas were 0.24‰ and 0.20‰, respectively, with no significant difference (OR = 1.20, 95%CI: 0.90–1.61). The prevalences of microtia-anotias for maternal age < 20, 20–24, 25–29, 30–34, and ≥ 35 were 0.36‰, 0.23‰, 0.21‰, 0.21‰, and 0.25‰, respectively, with no significant difference (*P* > .05, reference: 25–29).

Table 2 shows the prevalence details by sex, residence, and maternal age (Table 2).

**Table 2:** Prevalence of congenital malformations of the external ear by residence, sex, and maternal age.

Variables	Fetuses (n)	Microtia-anotia			Other CMEE			Total CMEE		
		n	Prevalence (‰,95%CI)	OR (95%CI)	n	Prevalence (‰,95%CI)	OR (95%CI)	n	Prevalence (‰,95%CI)	OR (95%CI)
Sex										
Male	448,288	118	0.26 (0.22–0.31)	1.57 (1.16–2.12)	600	1.34 (1.23–1.45)	1.21 (1.07–1.37)	718	1.60 (1.48–1.72)	1.26 (1.12–1.41)
Female	399,368	67	0.17 (0.13–0.21)	Reference	442	1.11 (1.00–1.21)	Reference	509	1.27 (1.16–1.39)	Reference
Unknown	99	0	-	-	0	-	-	0	-	-
Residence										
Urban	342,178	83	0.24 (0.19–0.29)	1.20 (0.90–1.61)	524	1.53 (1.40–1.66)	1.50 (1.32–1.69)	607	1.77 (1.63–1.92)	1.45 (1.29–1.62)
Rural	505,577	102	0.20 (0.16–0.24)	Reference	518	1.02 (0.94–1.11)	Reference	620	1.23 (1.13–1.32)	Reference
Maternal age (yr old)										
<20	13,711	5	0.36 (0.05–0.68)	1.76 (0.71–4.36)	19	1.39 (0.76–2.01)	1.12 (0.71–1.78)	24	1.75 (1.05–2.45)	1.22 (0.81–1.83)
20–24	118,531	27	0.23 (0.14–0.31)	1.10 (0.71–1.71)	123	1.04 (0.85–1.22)	0.84 (0.69–1.03)	150	1.27 (1.06–1.47)	0.88 (0.73–1.05)
25–29	357,582	74	0.21 (0.16–0.25)	Reference	441	1.23 (1.12–1.35)	Reference	515	1.44 (1.32–1.56)	Reference
30–34	243,649	50	0.21 (0.15–0.26)	0.99 (0.69–1.42)	308	1.26 (1.12–1.41)	1.03 (0.89–1.19)	358	1.47 (1.32–1.62)	1.02 (0.89–1.17)
≥35	114,282	29	0.25 (0.16–0.35)	1.23 (0.80–1.88)	151	1.32 (1.11–1.53)	1.07 (0.89–1.29)	180	1.58 (1.34–1.81)	1.09 (0.92–1.30)

CI = confidence intervals, CMEE = congenital malformations of the external ear, OR = crude odds ratio.

### 3.3. Epidemiology of CMEEs

Table 3 shows the epidemiology distribution of CMEEs. Males had a higher proportion of CMEEs than females (58.52% vs 41.48%), as well as microtia-anotias (63.78% vs 36.22%); There is no significant difference in the proportion of CMEEs between urban and rural areas (49.47% vs 50.53%), while rural areas had a higher proportion of microtia-anotias than urban areas (55.14% vs 44.86%); Most CMEEs occurred in maternal age 25–29 (41.97%) or 30–34 (29.18%); The proportions of CMEEs in maternal secondary school or below, senior school and university or above were 21.92%, 35.45%, and 42.62%, respectively; Most CMEEs occurred in first-born child (47.03%) or second-born child (45.80%); A total of 11.65% of CMEEs were premature infants, including 10.02% of infants with gestational age at termination of pregnancy 32–36 weeks, and 1.63% of infants with gestational age at termination of pregnancy 28 to 31 weeks; Most CMEEs were diagnosed by clinical examinations (92.34%); Most CMEEs were diagnosed postpartum (within 7 days) (96.25%); A total of 2.77% CMEEs end in deaths (Table 3).

**Table 3:** Epidemiology of congenital malformations of the external ear.

Variables	Microtia-anotia		Other CMEE		Total CMEE	
	n	Proportion (%)	n	Proportion (%)	n	Proportion (%)
Sex						
Male	118	63.78	600	57.58	718	58.52
Female	67	36.22	442	42.42	509	41.48
Residence						
Urban	83	44.86	524	50.29	607	49.47
Rural	102	55.14	518	49.71	620	50.53
Maternal age (yr old)						
<20	5	2.70	19	1.82	24	1.96
20–24	27	14.59	123	11.80	150	12.22
25–29	74	40.00	441	42.32	515	41.97
30–34	50	27.03	308	29.56	358	29.18
≥35	29	15.68	151	14.49	180	14.67
Maternal education level						
Secondary school or below	50	27.03	219	21.02	269	21.92
Senior school	70	37.84	365	35.03	435	35.45
University or above	65	35.14	458	43.95	523	42.62
Parity						
0	2	1.08	3	0.29	5	0.41
1 (first-born)	82	44.32	495	47.50	577	47.03
2 (second-born)	85	45.95	477	45.78	562	45.80
≥3	16	8.65	67	6.43	83	6.76
Gestational age at termination of pregnancy						
28–31 wk	4	2.16	16	1.54	20	1.63
32–36 wk	23	12.43	100	9.60	123	10.02
≥37 wk	158	85.41	926	88.87	1084	88.35
Diagnostic methods						
B-Ultrasound	10	5.41	20	1.92	30	2.44
Clinical examinations	163	88.11	970	93.09	1133	92.34
Other	12	6.49	52	4.99	64	5.22
Time of diagnosis						
Antepartum (28 wk or above)	13	7.03	33	3.17	46	3.75
Postpartum (within 7 d)	172	92.97	1009	96.83	1181	96.25
Perinatal deaths						
Yes	10	5.41	24	2.30	34	2.77
No	175	94.59	1018	97.70	1193	97.23
Total	185	100.00	1042	100.00	1227	100.00

CMEE = congenital malformations of the external ear.

## 4. Discussion

Overall, we have described the prevalence and epidemiology of CMEEs. Our study is the most recent systematic study on CMEEs in China, which may make some original contributions to the field.

There were several meaningful findings in this study. First, although many studies have reported the prevalence of microtia-anotias, there was a paucity of studies reporting the overall prevalence of CMEEs from birth defects surveillance. In this study, the prevalence of microtia-anotias was 0.22‰, consistent with the globally accepted (2.06 per 10,000 births, 95%CI: 2.02–2.10).^[29]^ Although the overall prevalence of CMEEs was not reported in several studies, the prevalence of various specific defects (including various specific CMEEs) was reported, from which we can estimate the overall prevalence of CMEEs. E.g., 1.74‰ in Hunan Province, China, 2005 to 2014^[19]^; 1.26‰ in Guilin City, China, 2018 to 2020^[30]^; 1.36‰ in Zhejiang Province, China, 2013 to 2017.^[31]^ It is also consistent with our study (1.21‰–1.80‰).

Second, from 2016 to 2020, the prevalence of birth defects showed a downward trend, while the prevalence of CMEEs showed no significant trend, which may be mainly related to prenatal screening and diagnosis of birth defects. Improvements in prenatal screening and diagnosis technologies caused more and more birth defects diagnosed early in pregnancy (before 28 weeks of gestation) and selective termination, which were not used to calculate the prevalence of birth defects. E.g., most Down syndrome are diagnosed and terminated in the second trimester, resulting in a low prevalence.^[32]^ Zhou et al reported that the prevalence of Down syndrome was 1.49 per 10,000 fetuses in Hunan Province, China, 2010 to 2020,^[5]^ which was significantly lower than the accepted prevalence (almost 1 in 600 live births).^[33]^ In contrast, most CMEEs were diagnosed postnatally, and few deaths were associated with CMEEs, which was the reason for the relatively stable prevalence.

Third, maternal age was not associated with the CMEEs, which was inconsistent with most specific defects.^[5,34–36]^ Different studies showed differences in the relationship between CMEEs and maternal age. E.g., several studies observed no clear relationship between maternal age and microtia-anotias or other CMEEs.^[5,37,38]^ In contrast, several studies have shown that the prevalence of microtia tends to increase with increasing maternal age,^[39,40]^ and Liu et al think the relationship between maternal age and microtia-anotias may be linked to some other factors, such as miscarriages.^[39]^ In general, the causes of CMEEs are poorly understood. Some previous studies supported that microtia-anotias may be related to environmental and genetic causes,^[25]^ whereas some studies supported that genetics had significant contributions.^[41–43]^ Our findings support the latter view, which requires in-depth research.

Fourth, CMEEs were more common in males and urban areas, consistent with most specific defects.^[5,44,45]^ However, the mechanism of urban-rural differences in the prevalence of CMEEs may differ from many other specific defects. Similar to the discussions above, urban-rural differences in the prevalence of birth defects (including many specific defects) may be related to etiologies, diagnosis, or surveillance,^[44]^ while CMEEs were not as most CMEEs were diagnosed postnatally. In addition, some studies found no difference in the prevalence of CMEEs (or microtia-anotias) between males and females,^[37,38]^ and several studies found the prevalence of CMEEs (or microtia-anotias) was higher in females than males.^[46]^ Moreover, some studies found racial differences in the prevalence of CMEEs (or microtia-anotias).^[26,29,47]^ It indicates that the difference in the prevalence of CMEEs between males and females, or urban areas and rural areas, may be mainly related to genetics, similar to the discussion above.

Fifth, we have described the epidemiology of CMEEs. Although we are unable to provide prevalence by demographic characteristics due to data limitations, it does present the basic features of CMEEs, such as clinical examinations diagnose most CMEEs, and males have a higher proportion of CMEEs than females, which may be useful for in-depth research in the future.

Some things could be improved in our study. First, we cannot provide the prevalence of CMEEs by demographic characteristics due to data limitations besides sex, residence, and maternal age. Second, many cases were combined with other defects, and some cases are syndromes. It requires detailed research. Third, some demographic characteristics, such as paternal age, are not included due to data limitations.

## 5. Conclusion

In summary, we have presented the epidemiology of CMEEs in Hunan Province, China. CMEEs were more common in males than females, in urban areas than rural areas, whereas there was no significant difference in prevalence of CMEEs by maternal age. We inferred that CMEEs may be mainly related to genetics, and the mechanism needs to be examined in the future.

## Acknowledgments

The authors thank all staff participating in the research from 2016 to 2020.

## Author contributions

**Conceptualization:** Xu Zhou, Junqun Fang.

**Data curation:** Xu Zhou, Junqun Fang, Xiaoli Wang, Haiyan Kuang, Jian He, Aihua Wang, Xinjun Hua.

**Formal analysis:** Xu Zhou.

**Funding acquisition:** Xu Zhou, Junqun Fang, Xiu Zeng, Shuxian Zeng.

**Methodology:** Xu Zhou, Xiu Zeng, Shuxian Zeng.

**Project administration:** Xu Zhou, Shuxian Zeng.

**Resources:** Shuxian Zeng.

**Supervision:** Xu Zhou, Xiu Zeng, Shuxian Zeng.

**Validation:** Xu Zhou, Xiu Zeng.

**Visualization:** Xu Zhou.

**Writing – original draft:** Xu Zhou, Xiaoli Wang, Haiyan Kuang.

**Writing – review & editing:** Xu Zhou, Junqun Fang, Xiu Zeng, Shuxian Zeng.
